# A novel pyroptosis-associated gene signature for immune status and prognosis of cutaneous melanoma

**DOI:** 10.7717/peerj.12304

**Published:** 2021-10-14

**Authors:** Zhengyuan Wu, Leilei Chen, Chaojie Jin, Jing Xu, Xingqun Zhang, Yi Yao

**Affiliations:** 1 Yuhang First People’s Hospital, Hangzhou, China; 2 The First Affiliated Hospital of Guangxi Medical University, Nanning, China

**Keywords:** Pyroptosis, Prognosis, Gene signature, Immune microenvironment, Immune status, Drug sensitivity, Cutaneous melanoma

## Abstract

**Background:**

Cutaneous melanoma (CM) is a life-threatening destructive malignancy. Pyroptosis significantly correlates with programmed tumor cell death and its microenvironment through active host-tumor crosstalk. However, the prognostic value of pyroptosis-associated gene signatures in CM remains unclear.

**Methods:**

Gene profiles and clinical data of patients with CM were downloaded from The Cancer Genome Atlas (TCGA) to identify differentially expressed genes associated with pyroptosis and overall survival (OS). We constructed a prognostic gene signature using LASSO analysis, then applied immune cell infiltration scores and Kaplan-Meier, Cox, and pathway enrichment analyses to determine the roles of the gene signature in CM. A validation cohort was collected from the Gene Expression Omnibus (GEO) database.

**Results:**

Four pyroptosis-associated genes were identified and incorporated into a prognostic gene signature. Integrated bioinformatics findings showed that the signature correlated with patient survival and was associated with tumor growth and metastasis. The results of Gene Set Enrichment Analysis of a risk signature indicated that several enriched pathways are associated with cancer and immunity. The risk signature for immune status significantly correlated with tumor stem cells, the immune microenvironment, immune cell infiltration and immune subtypes. The expression of four pyroptosis genes significantly correlated with the OS of patients with CM and was related to the sensitivity of cancer cells to several antitumor drugs. A signature comprising four genes associated with pyroptosis offers a novel approach to the prognosis and survival of patients with CM and will facilitate the development of individualized therapy.

## Introduction

Cutaneous melanoma (CM) is a destructive malignancy that threatens human health (Ekwueme et al., 2011). Among 287,723 new patients who were diagnosed with melanoma worldwide during 2018, 21.1% of them died of the disease (Bray et al., 2018). Metastatic CM increases the number of deaths associated with skin tumors (Finn, Markovic & Joseph, 2012). The 10-year overall survival (OS) rate is 75–98% for patients with stages I and II CM (Gershenwald et al., 2017), and 24–88% for those with stage IIIA to IIID CM. These data suggest that early diagnosis of CM is essential for favorable outcomes. Some theories are based on the notion that tumorigenesis and progression are related to skin pigmentation (Board PDQATE, 2002) and that pathogenesis is associated with acquired melanocytic nevi, family history, and genetic susceptibility (Gilchrest et al., 1999; Hawkes, Truong & Meyer, 2016), but the pathogenesis of CM remains unknown. Most previous studies have mainly focused on accurately diagnosing early CM. More recent studies have aimed to uncover new biomarkers to predict the prognosis of CM for personalized treatment. However, only a few clinically relevant biomarkers and tools for CM have been identified (Wang et al., 2019). Thus, biomarkers are needed that can help to diagnose and identify the prognosis of CM.

Pyroptosis is a type of lytic cell death characterized by bubble-like protrusions and cellular swelling (Kovacs & Miao, 2017). The gasdermin family is regarded as the main executor of pyroptosis (Broz, Pelegrín & Shao, 2020). During the process of pyroptosis, cells can form various vesicles, and gasdermin is multimerized and sheared, forming 10–20-nm pores in the cell membrane. As cell contents continuous flow out of membrane pores, cells produce apoptotic vesicle-like protrusions that gradually swell and rupture (Frank & Vince, 2019; Zhang et al., 2018). Pyroptosis plays a key role in the development and progression of cancer. Various pyroptotic factors, such as gasdermin family genes, proinflammatory cytokines, and inflammatory vesicles are significantly associated with tumorigenesis and metastasis (Kolb et al., 2014). Compared with cell apoptosis, pyroptotic cells can activate and release various danger-associated signaling cytokines to induce a powerful inflammatory response and immune system activation (Tang et al., 2020). The potent anti-tumor properties of pyroptosis are related to modulation of the immune microenvironment of tumors containing NK cells (Zhang et al., 2020b) and CD8+ T lymphocytes (Xi et al., 2019). Thus, the notion that pyroptosis is associated with tumors and anti-tumor progress is widely accepted, but its specific role in CM remains poorly understood.

Disease-specific biomarkers of CM have been identified using bioinformatics. However, few gene expression signatures have been identified that can predict the prognosis of patients with CM. Moreover, no systematic attempt has been made to identify specific pyroptosis-associated hub genes that correlate with cancer prognosis or progression. Here, candidate pyroptosis genes that were significantly related to CM prognosis were determined using differential gene expression and univariate Cox regression analysis. Thereafter, hub genes were characterized using the least absolute shrinkage and selection operator (LASSO) and a gene signature was constructed. The function and clinical significance of this gene signature was explored. We also systematically investigated the association between the pyroptosis gene signature and the immune microenvironment, types of immune infiltrates, cancer chemoresistance, and tumor stemness. To the best of our knowledge, the present report describes the first pyroptosis-associated risk signature for CM prognosis, which will provide novel insights into the diagnosis and prognosis of patients with CM.

## Materials and Methods

### Raw data acquisition

Table 1 shows clinical information and RNA sequences of 471 CM and one normal skin sample from The Cancer Genome Atlas (TCGA) database on June 30, 2020 (https://portal.gdc.cancer.gov). Transcriptome data for 812 normal skin samples were obtained from the Genotype-Tissue Expression database (GTEx; https://gtexportal.org/home/datasets). Clinical and gene expression data for 355 CM samples were downloaded from the Gene Expression Omnibus (GEO; https://www.ncbi.nlm.nih.gov/geo/) GSE59455 and GSE65904 cohorts for external validation. Batch effects were removed by Log2-transformation and normalization using the sva package in R (Xiao et al., 2020; Zhang et al., 2020a), and 146 protein domains for specific pyroptosis genes were collected from the GeneCards database (https://www.genecards.org) (Table S1).

**Table 1:** Details of datasets in this study.

Accession number	Number of samples		Country	Version
	Non-tumor skin tissue	Melanoma tissue		
TCGA	1	471	USA	2019
GTEx	812	/	/	2016
GSE59455	/	141	Australia	2018
GSE65904	/	214	Sweden	2020
Total	813	826		

### Prognostic gene signature construction

Differentially expressed genes (DEGs) were identified in the TCGA-CM cohort using the limma package. Genes with a |log2 fold change (FC)| > 1 and FDR < 0.05, were regarded as candidate DEGs as described (Li et al., 2020). Prognostic genes among all pyroptosis-related genes in the TCGA-CM cohort were identified using univariate Cox regression and the Kaplan–Meier survival package with a cutoff of *p* < 0.05. Overlapping DEGs and prognostic genes were considered as candidate pyroptosis genes and were visualized using the VennDiagram package. Thereafter, candidate pyroptosis genes were integrated into LASSO analysis using the glmnet package to identify hub pyroptosis genes and generate a gene risk signature. Patients with CM were categorized into low- and high-risk groups, and their risk scores were calculated as: ${\rm risk\; score} = {\Sigma expgenei*\beta i},$ where expgenei is the relative expression of pyroptosis gene i, and β is the regression coefficient (Bai et al., 2020). This formula and the same regression coefficients were also applied to the GEO validation cohorts and patients were stratified into two risk subgroups using the same median risk score.

### Evaluation of gene signature accuracy

The distribution of risk subgroups in the constructed signatures was explored by t-SNE and PCA analyses using the Rtsne and ggplot2 packages. Outcomes between two risk subgroups were also compared according to risk levels using the survival package. The predictive accuracy of the signature was calculated using the timeROC package in two databases. The implications of risk scores for clinical characteristics were evaluated by univariate and multivariate Cox regression analyses, and connections between risk scores and clinical characteristics in the TCGA-CM cohort were visualized using the ggpubr package.

### Function enrichment analyses

Kyoto Encyclopedia of Genes and Genomes (KEGG) enrichment was compared between two risk subgroups using Gene Set Enrichment Analysis (GSEA) software version 4.1. The biological functions of hub pyroptosis genes revealed by Gene Ontology (GO) and KEGG enrichment analyses were determined using the Database for Annotation, Visualization and Integrated Discovery (DAVID) version 6.8 (Huang, Sherman & Lempicki, 2009). Biological process (BP), molecular function (MF), and cellular component (CC) were applied to GO enrichment analyses. Values were considered statistically significant at FC > 1 and FDR *p* < 0.05.

### Immune response and tumor microenvironment analysis

Stromal and immune scores are commonly calculated to determine levels of stromal and immune cell tumor infiltration (Yoshihara et al., 2013). We examined the relationship between risk scored and stromal and immune scores using Spearman correlations. Connections between immune infiltration subtypes and risk scores were assessed by two-way ANOVA. Connections between risk scores and tumor stemness were analyzed using Spearman correlations to determine stem cell-like features of the gene signature.

### Chemotherapy sensitivity analysis

The NCI-60 database was obtained from the CellMiner interface (https://discover.nci.nih.gov/cellminer), and then 218 chemotherapy drugs (Table S2) were filtered after FDA standard certification and clinical laboratory verification. The sensitivity of hub pyroptosis genes to drugs and identification of the risk signature were determined by Pearson correlation analysis, and these results were visualized by using “limma”, “impute”, “ggplot2”, and “ggpubr” R packages.

## Results

### Prognostic DEGs screening

**Figure 1:**
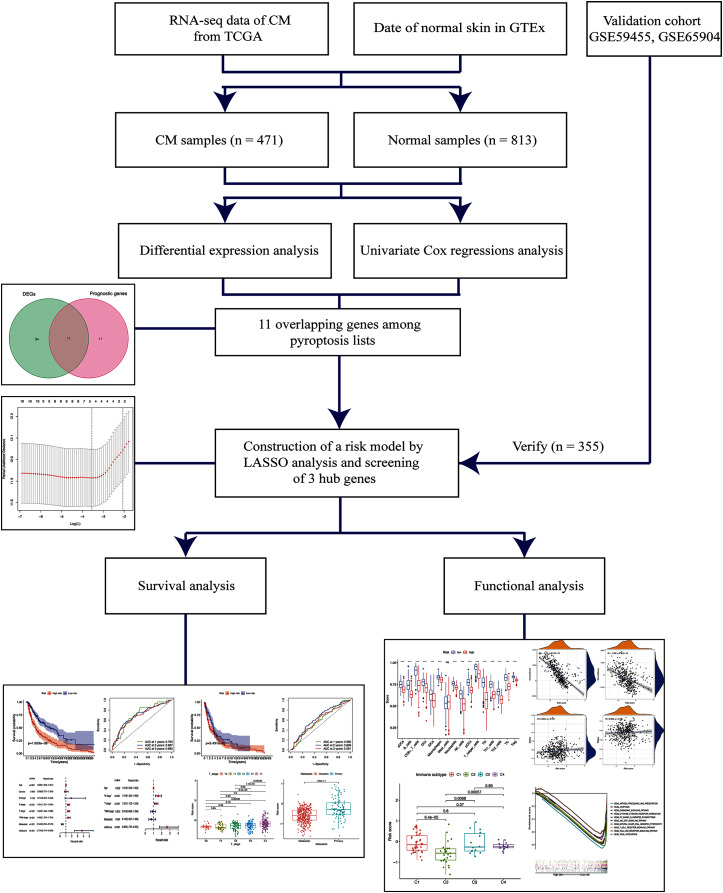
Schema of the study.

**Figure 2:**
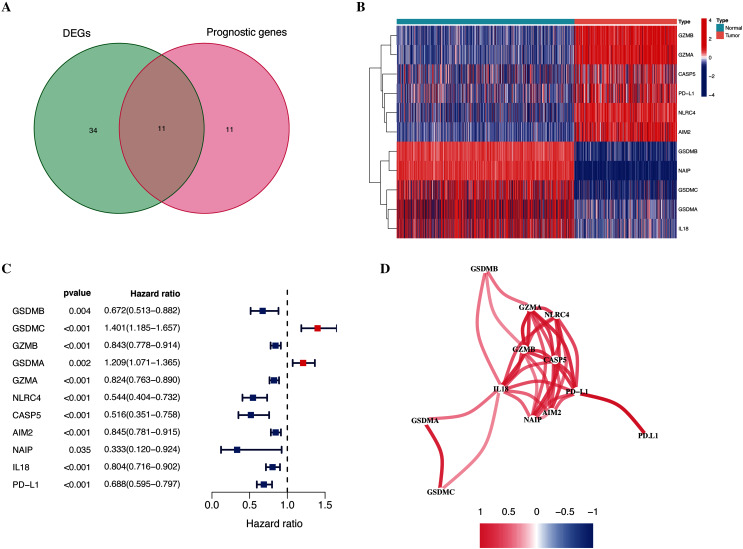
Identification of candidate prognostic DEGs in TCGA-CM cohort. (A) Venn diagram of pyroptosis-related genes determined by differential expression and univariate Cox analyses. (B) Heatmap of candidate prognostic DEGs in TCGA-CM cohort. (C) Forest plots of correlations between candidate prognostic DEGs and overall survival of patients in TCGA-CM cohort. (D) Correlation network of candidate prognostic DEGs.

As shown in Fig. 1, the differential expression analysis identified 45 pyroptosis-associated genes as DEGs within the TCGA dataset (Fig. 2A), among which, 11 were significantly associated with the OS of patients with CM (Fig. 2B). Thus, these 11 genes were identified as candidate prognostic DEGs. Univariate Cox analysis of the candidate DEGs verified their association with prognosis (Fig. 2C), and correlation analysis showed that they were connected to each other (Fig. 2D).

### Construction of a gene signature for patients with CM

**Figure 3:**
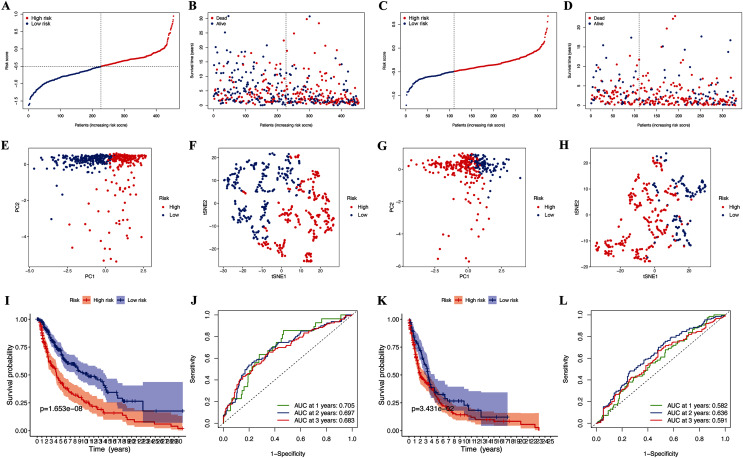
Prognostic analysis of risk signature. Risk score distribution (A, C) and survival status (B, D) of TCGA-CM and GEO cohorts, respectively. PCA plot (E) and t-SNE (F) analysis of TCGA-CM cohort. PCA plot (G) and t-SNE (H) analysis of validation cohort. (I) Survival curve of the TCGA cohort. (J) TimeROC curves to forecast overall survival of patients from TCGA-CM cohort. (K) Survival curve of validation cohort. (L) TimeROC curves to forecast overall survival of patients in validation cohort.

Further LASSO analysis identified 11 prognostic DEGs, and the four hub pyroptosis genes, gasdermin C (GSDMC), granzyme A (GZMA), absent in melanoma 2 (AIM2), and programmed death-ligand 1 (PD-L1), with which the risk signature was constructed (Table 2, Fig. S1). Patients in the TCGA-CM (Figs. 3A and 3B) and GEO (Figs. 3C and 3D) cohorts were divided into low- and high-risk subgroups based on their median risk scores. The results of the t-SNE and PCA analyses indicated that patients in the two risk subgroups were distributed in different directions in the TCGA (Figs. 3E and 3F) and GEO (Figs. 3G and 3H) cohorts.

**Table 2:** Four prognosis-associated pyroptosis genes in the TCGA-CM cohort were identified by LASSO analysis.

Gene name	Univariate Cox regression analysis		Differential gene expression analysis		LASSO coefficient
	HR	*P* value	LogFC	*P* value	
GSDMC	1.4012	7.82E−05	−2.9217	6.80E−123	0.2758
GZMA	0.8241	7.49E−07	3.5244	2.01E−179	−0.0699
AIM2	0.8455	3.12E−05	3.3358	5.85E−146	−0.0526
PD-L1	0.6883	5.77E−07	1.1542	1.84E−65	−0.1766

### Associations between clinical characteristics and risk scores of patients with CM

The OS of high-risk patients with CM decreased in the TCGA cohort (Fig. 3I). Receiver operating characteristics (ROC) curves indicated that the predictive accuracy of our risk signature was moderate at years 1 (ROC = 0.705), 2 (ROC = 0.697), and 3 (ROC = 0.683) years (Fig. 3J). The results were the same for patients in the GEO cohorts (Figs. 3K and 3L), which confirmed our risk signature as a sensitive and specific predictor of the OS of patients with CM.

**Figure 4:**
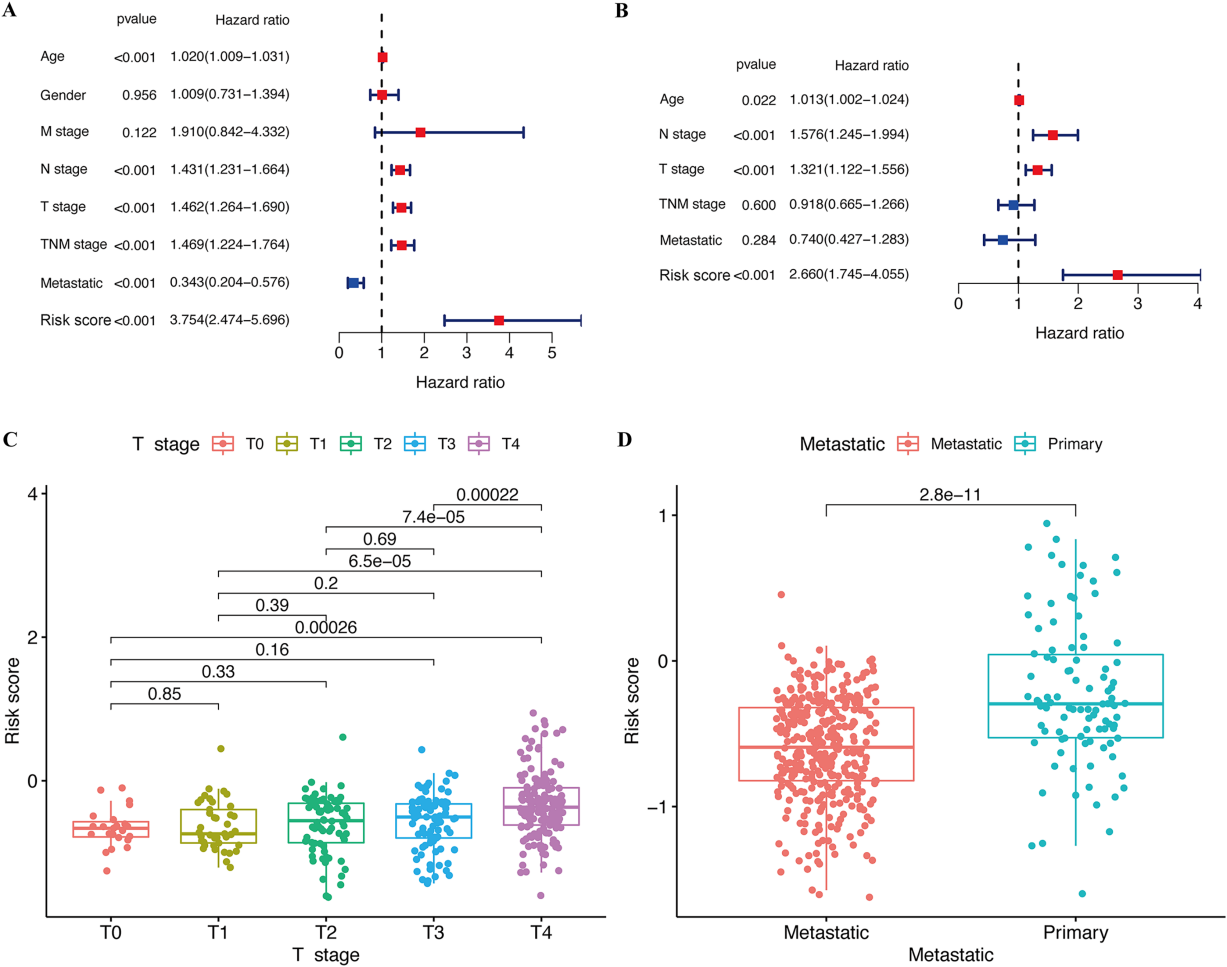
Associations between risk signature and clinicopathological factors. Univariate (A) and multivariate Cox (B) regression of clinicopathological features in TCGA-CM cohort. Correlations between risk scores and T stage (C) and metastatic capacity (D) in TCGA-CM cohort.

The results of the multivariate and univariate Cox regression analyses revealed that the risk score was an independent prognostic factor for patients in the TCGA cohort (Fig. 4B), whereas the risk signature was associated with prognosis (Fig. 4A). The gene signature was significantly associated with tumor T stage and metastatic state (Table S3). Patients with higher T stages had significantly higher risk scores (*p* < 0.05, Fig. 4C). Meanwhile, primary melanoma was significantly associated with higher risk scores (*p* < 0.05, Fig. 4D). These findings showed that our risk signature was associated with the development of CM.

### Associations of selected hub pyroptosis genes in CM

**Figure 5:**
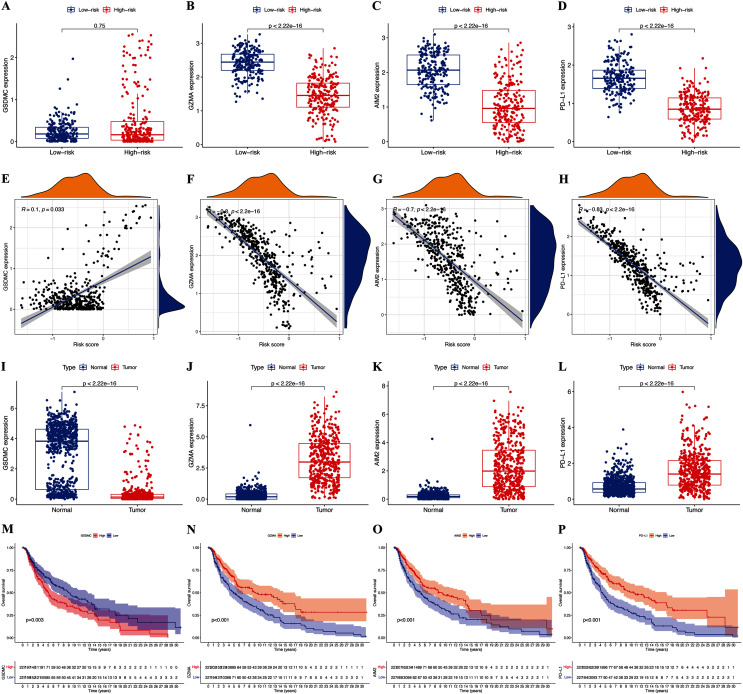
Roles of pyroptosis genes in risk signature and CM prognosis. Expression of GSDMC (A), GZMA (B), AIM2 (C), and PD-L1 (D) genes in risk subgroups. Correlations between risk signature and GSDMC (E), GZMA (F), AIM2 (G), and PD-L1 (H) genes. Expression of GSDMC (I), GZMA (J), AIM2 (K), and PD-L1 (L) genes in CM. Kaplan-Meier curves of TCGA-CM cohort verify prognostic value of GSDMC (M), GZMA (N), AIM2 (O), and PD-L1 (P).

The expression of hub pyroptosis genes (except GSDMC) was significantly decreased in the high-risk, compared with the low-risk subgroup (Fig. 5A–5D). The expression of GZMA, AIM2, and PD-L1 was associated negatively (*p* < 0.05; Figs. 5F–5H), and that of GSDMC was associated positively (*p* < 0.05; Fig. 5E) with risk scores. These results were consistent with the Kaplan-Meier findings, which revealed that the prognosis of patients was associated positively with expression of the GZMA, AIM2, and PD-L1 genes (*p* < 0.05; Figs. 5N–5P) and negatively with that of GSDMC (*p* < 0.05; Fig. 5M). Abundant expression of the prognostic genes GZMA, AIM2, and PD-L1, and less GSDMC expression were founded in samples from patients with CM than healthy controls (Figs. 5I–5L).

### Tumor microenvironment and immune response

**Figure 6:**
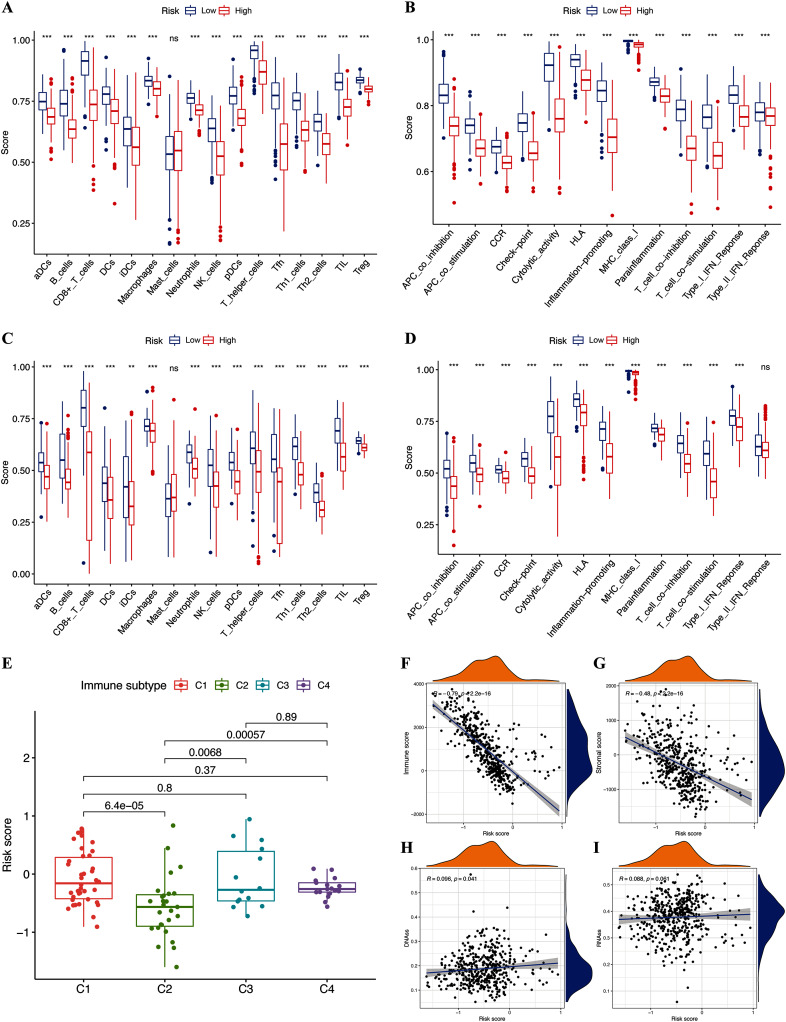
Potential role of risk signature in CM immune status and tumor stemness. Boxplots of scores of immune cells (A) and immune-associated functions (B) in risk subgroups of TCGA-CM cohort. Boxplots of scores for immune cells (C) and immune-associated functions (D) in risk subgroups of validation cohort. Associations between risk signature and immune infiltration subtypes (E) and immune scores (F), stromal scores (G), DNAss (H), and RNAss (I).

We assessed the relationship between immune status and risk signature in the TCGA-CM cohort. Nearly all immune cell subpopulations, related pathways, and functions were significantly reduced in the high-risk subgroup (*p* < 0.05; Figs. 6A and 6B). Only mast cell scores did not differ between the two subgroups. The results of the GEO cohort were similar (Figs. 6C and 6D). Immune infiltrates corresponding to human tumor suppression and promotion, respectively (Tamborero et al., 2018), namely C1 (wound healing), C2 (INF-g dominant), C3 (inflammatory), and C4 (lymphocyte depleted), were applied to understand the connection between immune components and the risk signature. The calculated risk score significantly declined in the C2 immune subtype (Fig. 6E). The C2 immune subtype was also significantly associated with upregulated gene expression of GZMA, AIM2, and PD-L1 (Figs. S2B–S2D). In contrast, GSDMC was expressed at significantly lower levels in the C2 subtype (Fig. S2A).

The immune microenvironment (including immune and stromal scores) and tumor stemness (including RNA stemness score and DNA methylation profiles) are key regulators of tumor progression. The risk signature correlated significantly and negatively with immune and stromal scores (*p* < 0.05; Figs. 6F and 6G), but positively with DNA methylation profiles (DNAss; *p* < 0.05; Figs. 6H and 6I). Expression of the pyroptosis genes except for GSDMC, correlated positively with immune and stromal scores in patients with CM (*p* < 0.05; Figs. S3I–S3P), and negatively with CM stemness scores, whereas GSDMC correlated negatively only with RNAss scores (*p* < 0.05; Figs. S3A–S3H).

### Functional enrichment analysis

**Figure 7:**
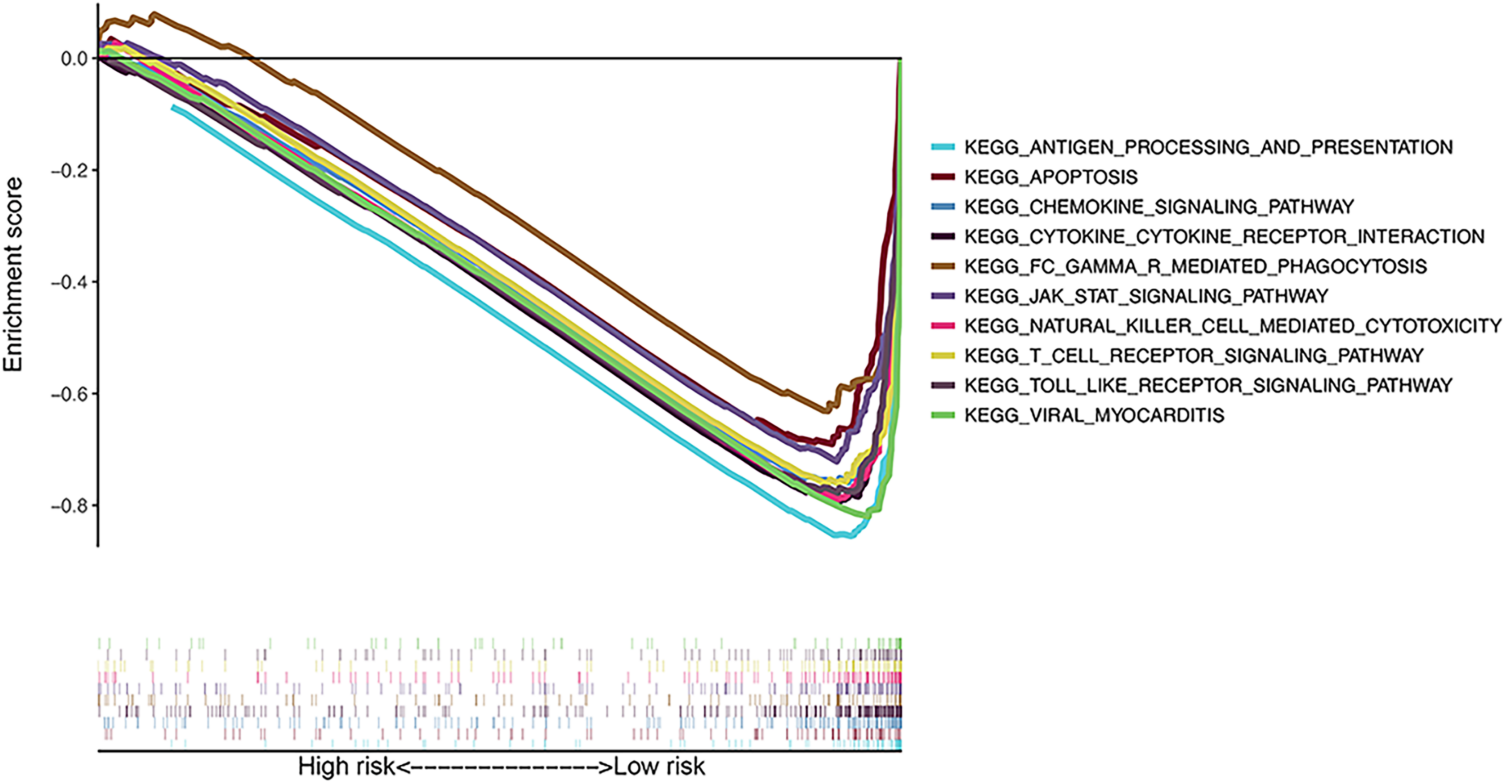
Gene Set Enrichment Analysis of top 10 enriched pathways in risk signature.

The results of KEGG pathway enrichment analysis of the risk subgroups using GSEA software revealed 53 significantly enriched pathways in the low-risk subgroup (FDR < 0.05; Table S4), including antigen processing and presentation, apoptosis, and chemokine signaling pathway (Fig. 7). Meanwhile, several immune-related pathways, such as natural killer cell-mediated cytotoxicity, T cell receptor signaling, and Toll-like receptor signaling pathways were also enriched in the risk signature.

GO and KEGG enrichment were also analyzed to determine the biological functions of the four pyroptosis genes. The results of the GO enrichment analysis showed that hub genes were predominantly augmented in several BPs, such as pyroptosis, positive regulation of response to biotic stimulus, and regulation of deoxyribonuclease activity (Figs. S4A and S4B). Hub pyroptosis genes were also enriched in inflammasome complex, immunological synapse, and recycling endosome membrane in the CC category, as well as phosphatidylinositol-4-phosphate binding, phosphatidylserine binding, and phosphatidylinositol-4,5-bisphosphate binding in the MF category. The results of the KEGG enrichment analysis revealed augmented pyroptosis genes in the cytosolic DNA-sensing pathway (Figs. S4C and S4D). We then investigated the specific mechanisms of the pyroptosis genes in CM.

### Relationships between pyroptosis genes and chemotherapy drug sensitivity

**Figure 8:**
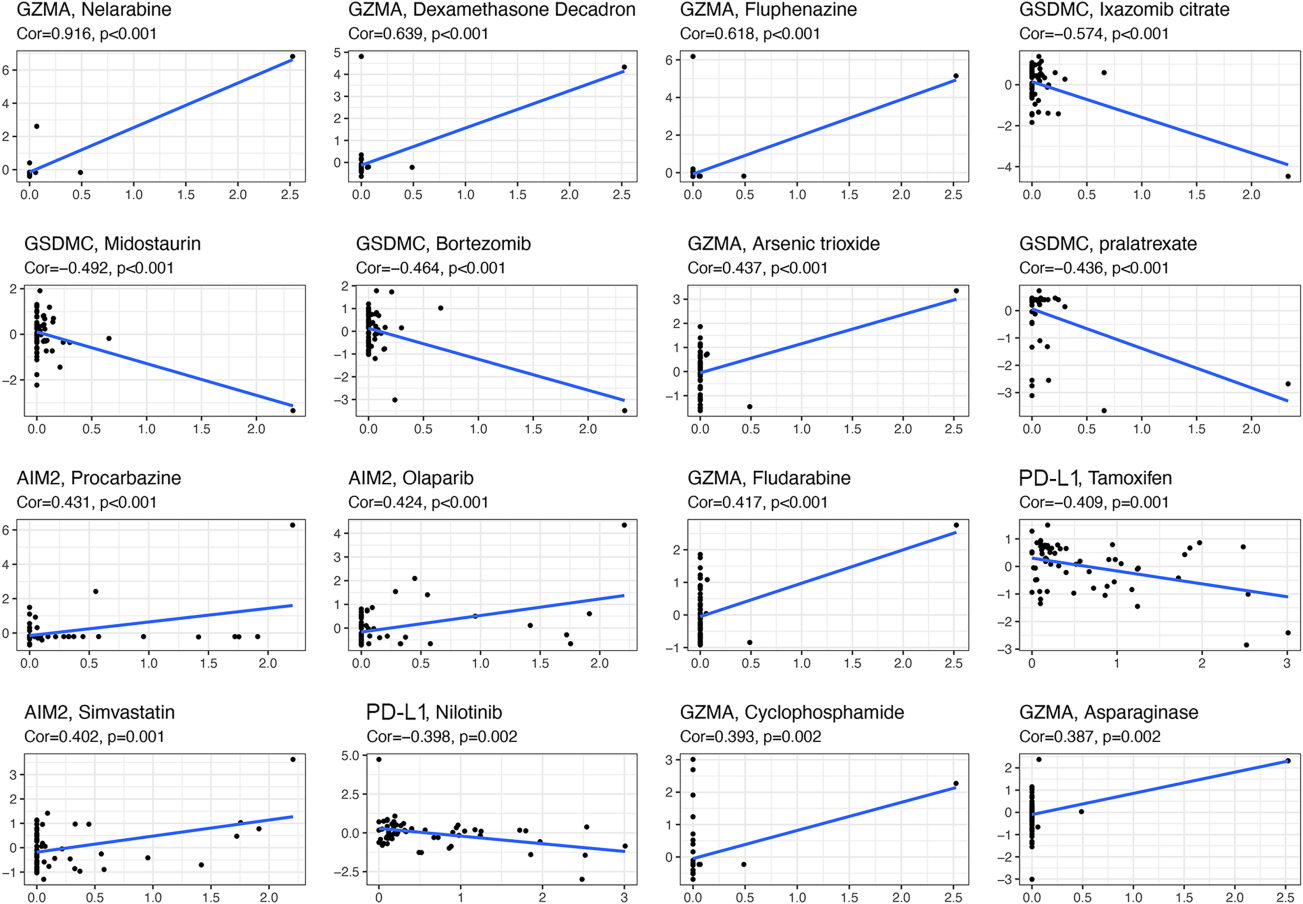
Scatter plots of top 16 classes of associations between pyroptosis genes and drug sensitivity.

All the prognostic genes correlated with sensitivity to several drugs (*p* < 0.05, Table S5). For example, GZMA and AIM2 gene expression in tumor cells was positively associated with increased drug sensitivity to nelarabine, dexamethasone decadron, fluphenazine, arsenic trioxide, procarbazine, olaparib, fludarabine, simvastatin, cyclophosphamide, and asparaginase (Fig. 8). In contrast, increased GSMDC and PD-L1 gene expression was positively associated with increased drug resistance of tumor cells to ixazomib citrate, midostaurin, bortezomib, pralatrexate, tamoxifen, and nilotinib.

## Discussion

Despite the establishment of next-generation sequencing technology and the discovery of various biomarkers for melanoma, more novel markers that are more closely associated with the early detection and predicted outcomes of CM are still needed. The prognostic performance of pyroptosis, which significantly correlates with programmed cell death, is good in ovarian cancer (Ye, Dai & Qi, 2021). However, its role in CM has not been systemically studied. Recently, a pyroptosis-associated gene signature for CM was constructed (Ju et al., 2021), however, this study only selected 20 pyroptosis-associated genes for risk model construction, and they did not conduct in-depth research on the mechanisms related to CM progression. In this study, 146 pyroptosis-associated gene lists were downloaded from the public database, which provides an excellent chance to identify more potential molecular markers. Meanwhile, the differential expression analysis and univariate Cox analysis were firstly constructed to screen these genes, which might also help to discover more specific prognostic markers to screen for innovative treatment targets. Like signatures associated with immune checkpoint (Tian et al., 2020), lncRNAs (Liu et al., 2019), ferroptosis (Luo & Ma, 2021), and hypoxia (Shou et al., 2020), our risk signature also had high predictive accuracy for the OS of patients with CM. We also analyzed whether immune status, tumor microenvironment, immune components, tumor stemness, and chemotherapeutic drug sensitivity corresponded to the gene signature and pyroptosis genes, and found that our risk signature conferred advantages compared with those described above.

We systematically investigated 146 pyroptosis-related genes, and the differential expression and univariate Cox regression analyses identified prognostic DEGs that were associated with OS. The LASSO analysis identified GSDMC, GZMA, AIM2, and PD-L1, as hub pyroptosis genes, and we constructed a novel and reliable predictive signature for the OS of patients with CM. The risk signature predicted the prognosis of patients with moderate accuracy. This result may be related to the role of identified genes in CM prognosis. For example, GSDMC is not only connected with pyroptosis, but also affects CM hypoxic microenvironment. Hypoxia-associated gene risk signature, which including GSDMC, also show satisfactory prognostic effects in melanoma (Shou et al., 2020). Furthermore, we also considered the role of the signature in CM progression by assessing the relationship between the clinical features of patients and risk signatures. Our signature correlated with metastasis and T stage. The clinicopathological parameter tumor (T) stage is widely applied for clinical tumor evaluation. However, increasing evidence indicates that the T stage is unsuitable for comprehensively elucidating tumor behavior (Yan et al., 2019). Compared with the T stage, our risk signature not only had similar prognostic value, but it can also predict the growth and metastatic potential of CM.

With respect to the pyroptosis genes, the expression of GZMA, AIM2, and PD-L1 was upregulated, whereas that of GSDMC was downregulated in CM. All genes that were negatively associated with risk scores correlated positively with the OS of patients with CM, whereas results for the GSDMC gene were the opposite. These results confirmed a relationship between the risk signature and outcomes of CM. The tumor oncogene, GSDMC, can significantly promote cell proliferation and tumorigenesis in colorectal carcinogenesis and lung adenocarcinoma (Miguchi et al., 2016; Wei et al., 2020). Furthermore, the present findings were consistent with that fact that GSDMC can be a critical hypoxia-related risk factor in predicting the prognosis of melanoma with a calculated HR > 1 (Shou et al., 2020). However, the specific role of GSDMC in CM has not been assessed. Granzymes are a family of homologous serine proteases that can induce apoptosis in various tumor cells (Anthony et al., 2010). Granzymes also encode the genes of some virtual granzymes and components of immunocytes, that are part of the cancer immune microenvironment (Wu et al., 2021). Among human granzymes, GZMA is also pivotal for the activation of immunocytes in tumors (Romero et al., 2020; Rooney et al., 2015). By interacting with immune cells, GZMA can exert suppressive effects in some tumors, such as human B-lymphomas and bovine host leukocytes infected with *Theileria annulata* (Rchiad et al., 2020). This function was confirmed in the present study, as GZMA was positively associated with CM prognosis. Programmed death ligand-1 (CD274), is enriched mainly in antigen-presenting cells and tumor cell surfaces and rarely in normal samples, which makes it differentially functional with other co-inhibitory pathways (Dong et al., 2002). In some tumors, PD-L1 functions as an immune-inhibitory checkpoint cytokine that contributes to the inhibition of T cell-mediated immune responses. The immune escape of tumor cells and exhaustion of T cells can be promoted by PD-L1 binding to its receptor, PD-1. In addition, PD-L1 can also transmit anti-apoptotic signals to cancer cells and protect them from apoptosis (Wang et al., 2016). However, positive PD-L1 expression also correlates with better clinical outcomes of some tumor types, such as melanoma. Clinical trials have shown that PD-1/PD-L1 pathway-targeted monoclonal antibodies have resulted in impressive outcomes in patients with CM by preventing inhibition of the PD-L1 pathway and enhancing T cell functions (Allison, 2015; Ohaegbulam et al., 2015). Thus, PD-L1 is regarded as a selective antitumor target for therapy against CM. Absent in melanoma 2 (AIM2), which belongs to the Pyrin and HIN domain (PYHIN) family, is the first inflammasome with a defined ligand dsDNA. With respect to its tumor-modulating effects, AIM2 was initially identified as a melanoma tumor suppressor gene (DeYoung et al., 1997), which the present study confirmed. Notably, AIM2 exerts anti-tumorigenic effects in colon cancer (Choubey, 2016), nasopharyngeal carcinoma (Chen et al., 2012), and prostate cancer (Ponomareva et al., 2013), and pro-tumorigenic effects in cutaneous squamous carcinoma (Farshchian et al., 2017), HPV-associated cervical cancer (Milutin Gašperov et al., 2014), and non-small cell lung cancer (Kong et al., 2015). The specific mechanisms of action of AIM2 in these cancers remain obscure. Despite the fact that PD-L1 and AIM2 are associated with CM development, to date, the prognostic value of other pyroptosis genes in CM have not been assessed. Therefore, our findings might provide a novel perspective of the relationship between pyroptosis and CM prognosis and lead to the identification of valuable pyroptosis-connected biomarkers for personalized treatment.

The results of the GSEA analysis revealed significantly enriched immune-related pathways, namely natural killer cell-mediated cytotoxicity, T cell receptor signaling pathway, and Toll-like receptor signaling pathway, in the risk signature. The results of the GO and KEGG analyses revealed that the pyroptosis genes were also significantly associated with the immune functions of T cell proliferation and T cell apoptotic process. Thus, we speculate that the prognostic role of pyroptosis genes and risk signature is associated with immune progress. We investigated the potential mechanism of the risk signature in CM prognosis using several immune-associated analyses. Almost all immune cells were poorly infiltrative and immune functions were inadequately executed or inhibited in the high-risk subgroup. Considering the critical role of these immune cells in stimulating anti-tumor immunity (Shankaran et al., 2001), the anti-tumor immunity of high-risk patients with CM was probably significantly reduced. The ESTIMATE algorithm also revealed that scores of stromal and immune cells negatively correlated with risk scores, confirming that immune cells were poorly infiltrative in the high-risk subgroup. On the contrary, positive connections between pyroptosis genes and stromal and immune scores suggested that these genes participate in immune cell infiltration, play roles in stroma-associated activities, or are secreted by stromal cells. However, the specific connections between pyroptosis genes, risk signatures, and immune cells warrant further investigation.

We investigated the roles of the gene signature and pyroptosis genes in the immune infiltration type to improve understanding of the connection between immune components and CM. The results showed that C2 was significantly associated with a low risk score, low GSDMC expression, and high GZMA, AIM2, and PD-L1 expression. Considering the roles of the risk signature and pyroptosis genes in the OS of patients with CM, we concluded that C2 might protect against CM and inhibit tumor development.

Cancer stem cell-like cells (CSCs) comprise another important tumor growth promotion mechanism due to their ability to self-renew and invade. These cells are also the main reason for resistance to chemotherapeutics (Huang et al., 2010; Schonberg et al., 2014). The present study showed that the pyroptosis genes GZMA, AIM2, and PD-L1 were associated negatively, whereas the risk signature was associated positively with tumor stem cell scores. These findings confirmed that our gene signature is a risk characteristic, and that the GZMA, AIM2, and PD-L1 genes might inhibit CM. The CM risk factor, GSDMC, was negatively associated with RNAss, indicating that GSDMC promotes the proliferation of tumor cells and inhibits the differentiation of tumor stem cells through different pathways. However, the specific mechanisms require further exploration.

This study has several limitations. The retrospective design means that prospective studies are needed to confirm the results. More experimental studies are required to confirm the results we uncovered through bioinformatics. Mechanistic insight into the pyroptosis genes and their connections with the development of CM also awaits investigation.

## Conclusion

In conclusion, this study identified a novel pyroptosis-associated prognostic gene signature comprising four hub pyroptosis-associated genes with potent predictive accuracy. The gene signature was valuable in immune cell infiltration, immune functions, tumor microenvironment, immune components, and the drug sensitivity of CM. To the best of our knowledge, this is the first pyroptosis-connected signature that predicts the outcome of patients with CM. Our method of understanding the specific effects of pyroptosis in CM will significantly contribute to the literature because our prognostic signature provides novel perspectives on the outcomes of CM and individualized treatment.

## Supplemental Information

10.7717/peerj.12304/supp-1 Supplemental Information 1 LASSO findings of factors and construction of risk signature. (A) LASSO coefficient profiles of candidate prognostic DEGs. (B) Selection of penalty parameter (λ) in LASSO model.

10.7717/peerj.12304/supp-2 Supplemental Information 2 Expression of pyroptosis genes GSDMC (A), GZMA (B), AIM2 (C), and PD-L1 (D) in different immune infiltrate subtypes.

10.7717/peerj.12304/supp-3 Supplemental Information 3 Potential roles of genes in CM immune status and tumor stemness. Associations between GSDMC (A, E), GZMA (B, F), AIM2 (C, G), PD-L1 (D, H) genes and DNAss and RNAss, respectively. Associations between GSDMC (I, M), GZMA (J, N), AIM2 (K, O), PD-L1 (L, P) genes and immune andstromal scores, respectively.

10.7717/peerj.12304/supp-4 Supplemental Information 4 Functional enrichment analysis. (A, B) GO enrichment terms of hub pyroptosis genes in CC, BP, and MF. (C, D) KEGG enrichment terms of hub pyroptosis genes.

10.7717/peerj.12304/supp-5 Supplemental Information 5 The 146 pyroptosis-related genes.

10.7717/peerj.12304/supp-6 Supplemental Information 6 The 218 chemotherapy drugs of FDA approved.

10.7717/peerj.12304/supp-7 Supplemental Information 7 Baseline characteristics of CM patients in different subgroups.

10.7717/peerj.12304/supp-8 Supplemental Information 8 All significant KEGG pathways associated with risk subgroups were analyzed by GSEA.

10.7717/peerj.12304/supp-9 Supplemental Information 9 Association between the identified pyroptosis genes expression and chemotherapy drug sensitivity.

## Additional Information and Declarations

### Data Availability

The following information was supplied regarding data availability: The datasets analysed during the current study are available at: - TCGA: https://portal.gdc.cancer.gov; GEO database: https://www.ncbi.nlm.nih.gov/geo/query/acc.cgi?acc= - GTEx: https://xenabrowser.net/datapages/?cohort=GTEX&removeHub=https%3A%2F%2Fxena.treehouse.gi.ucsc.edu%3A443 - GEO: GSE65904. (https://portal.gdc.cancer.gov, https://gtexportal.org/home/datasets, https://www.ncbi.nlm.nih.gov/geo/).
