# Supplementary figures and images for: A novel pyroptosis-associated gene signature for immune status and prognosis of cutaneous melanoma
Source: PeerJ. 2021 Oct 14;9:e12304. doi: 10.7717/peerj.12304 (PMC8520690; doi:10.7717/peerj.12304)

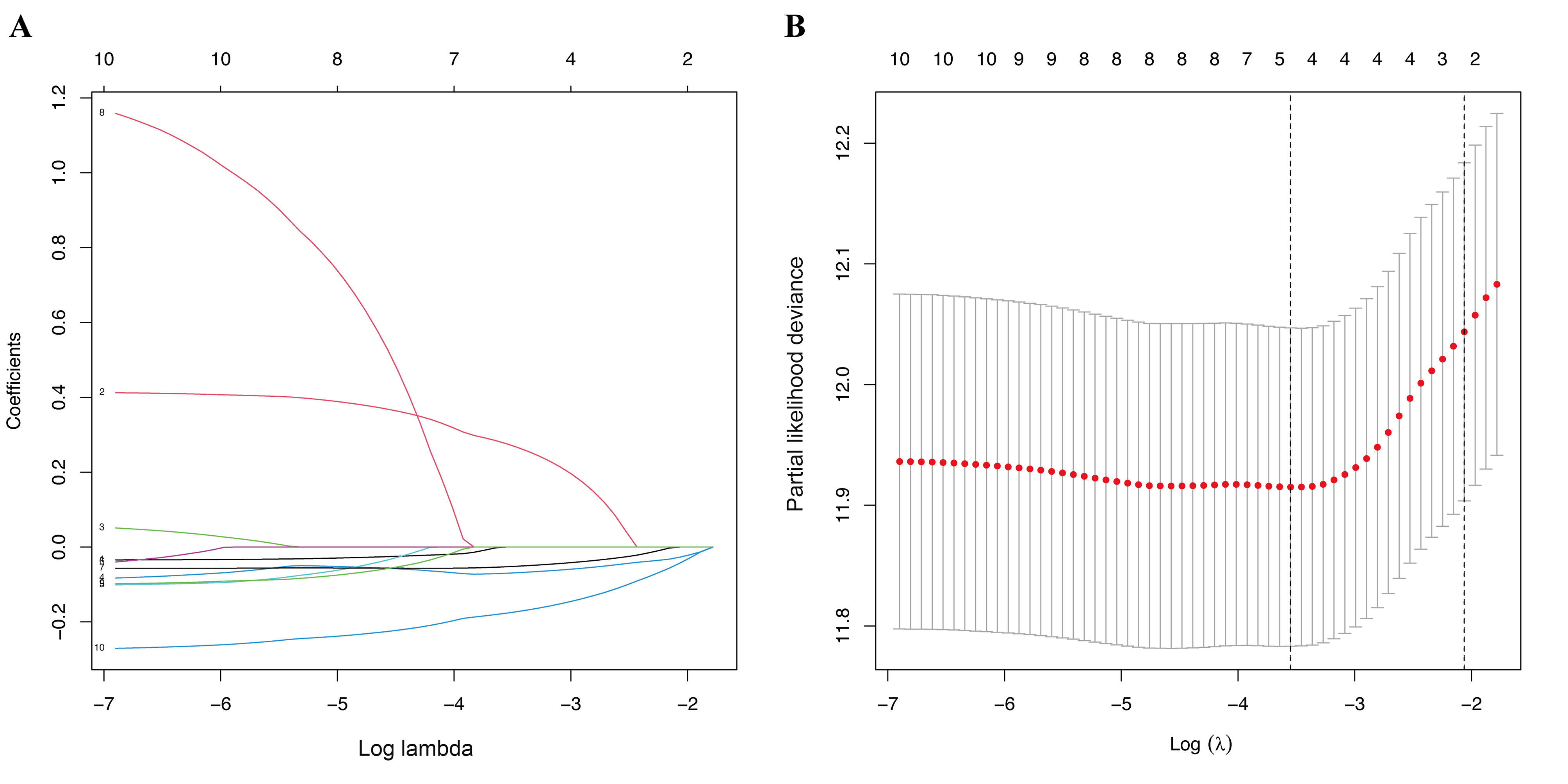

Supplement: Supplemental Information 1 — (A) LASSO coefficient profiles of candidate prognostic DEGs. (B) Selection of penalty parameter (λ) in LASSO model. [file peerj-09-12304-s001.png]

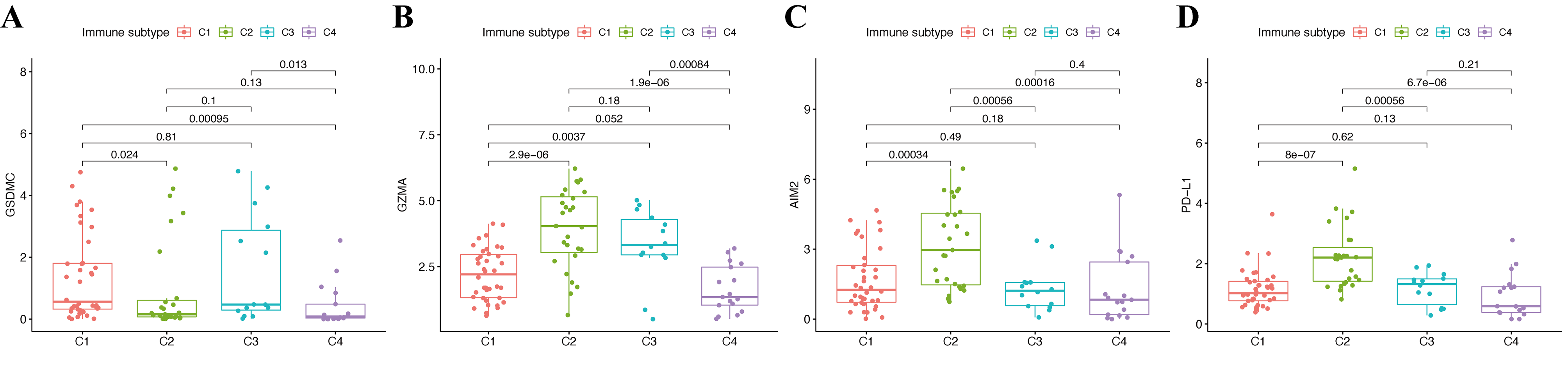

Supplement: Supplemental Information 2 [file peerj-09-12304-s002.png]

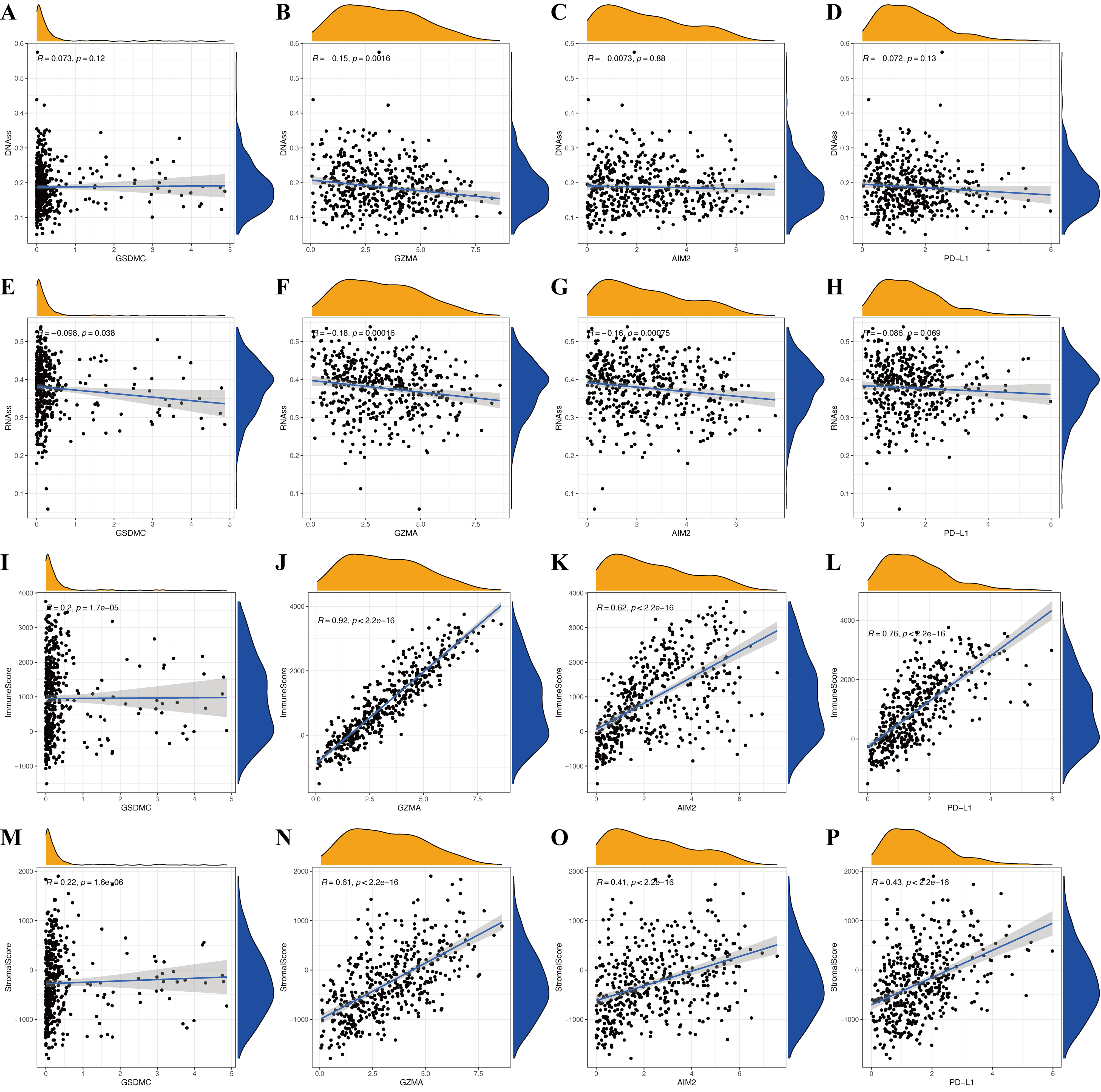

Supplement: Supplemental Information 3 — Associations between GSDMC (A, E), GZMA (B, F), AIM2 (C, G), PD-L1 (D, H) genes and DNAss and RNAss, respectively. Associations between GSDMC (I, M), GZMA (J, N), AIM2 (K, O), PD-L1 (L, P) genes and immune andstromal scores, respectively. [file peerj-09-12304-s003.png]

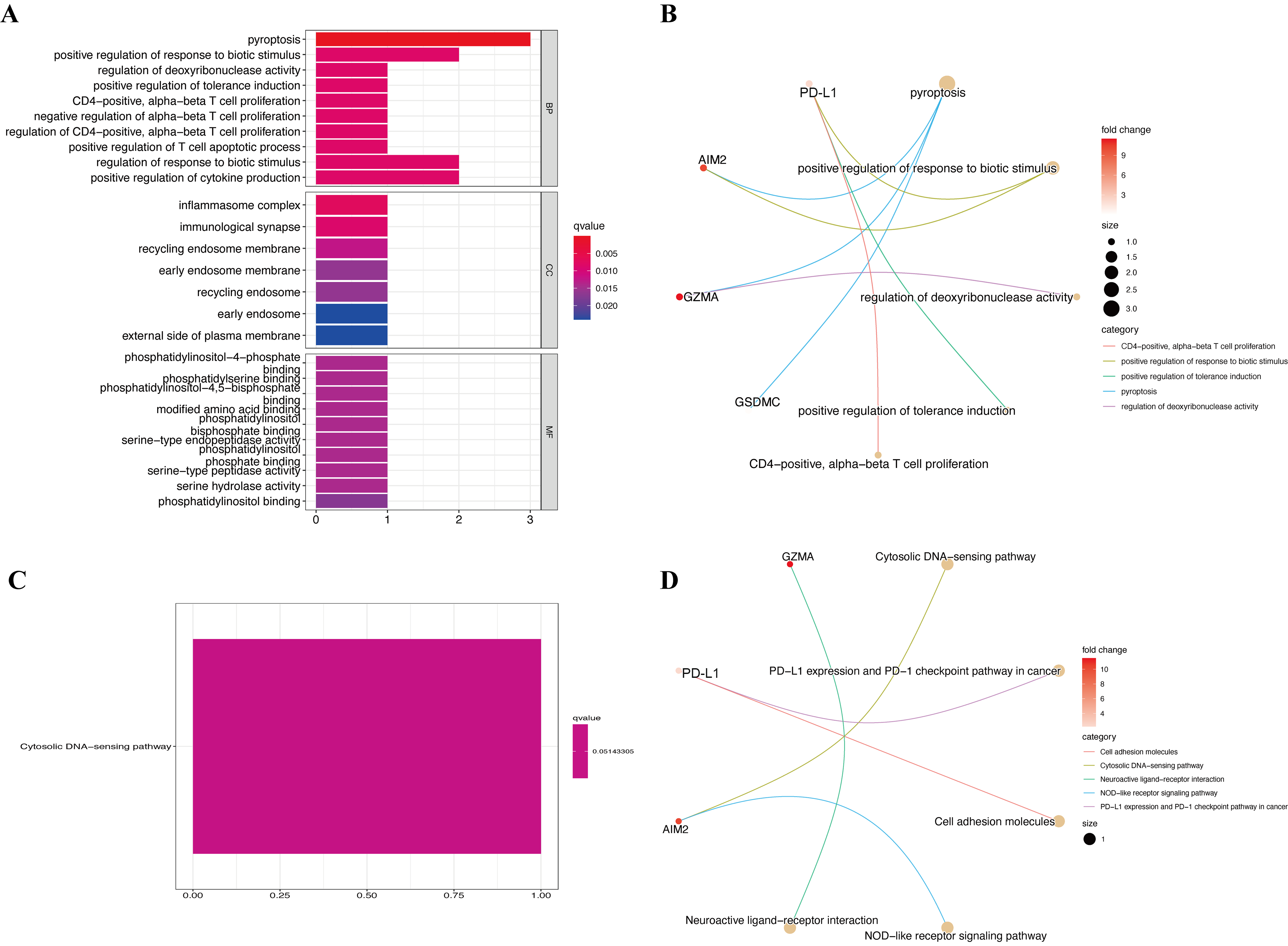

Supplement: Supplemental Information 4 — (A, B) GO enrichment terms of hub pyroptosis genes in CC, BP, and MF. (C, D) KEGG enrichment terms of hub pyroptosis genes. [file peerj-09-12304-s004.png]
