# Supplementary material for: A novel pyroptosis-associated gene signature for immune status and prognosis of cutaneous melanoma
Source: PeerJ. 2021 Oct 14;9:e12304. doi: 10.7717/peerj.12304 (PMC8520690; doi:10.7717/peerj.12304)
Supplement: Supplemental Information 5 [file peerj-09-12304-s005.docx]

**Supplementary Table 1**

The 146 pyroptosis-related genes.

| Gene symbol | Gene description | Gene symbol | Gene description | | | | | |
| --- | --- | --- | --- | --- | --- | --- | --- | --- |
| GSDMD | Gasdermin D | MRE11 | MRE11 Homolog, Double Strand Break Repair Nuclease | | | | | |
| GSDME | Gasdermin E | PARP1 | Poly(ADP-Ribose) Polymerase 1 | | | | | |
| NLRP3 | NLR Family Pyrin Domain Containing 3 | CTSG | Cathepsin G | | | | | |
| CASP1 | Caspase 1 | GBP5 | Guanylate Binding Protein 5 | | | | | |
| CASP4 | Caspase 4 | NLRP7 | NLR Family Pyrin Domain Containing 7 | | | | | |
| GSDMB | Gasdermin B | MKI67 | Marker Of Proliferation Ki-67 | | | | | |
| GSDMC | Gasdermin C | IL36G | Interleukin 36 Gamma | | | | | |
| IL1B | Interleukin 1 Beta | IL36B | Interleukin 36 Beta | | | | | |
| GZMB | Granzyme B | CPTP | Ceramide-1-Phosphate Transfer Protein | | | | | |
| NLRP1 | NLR Family Pyrin Domain Containing 1 | BNIP3 | BCL2 Interacting Protein 3 | | | | | |
| GSDMA | Gasdermin A | ANO6 | Anoctamin 6 | | | | | |
| GZMA | Granzyme A | MIR103A2 | MicroRNA 103a-2 | | | | | |
| NLRC4 | NLR Family CARD Domain Containing 4 | MIR103A1 | MicroRNA 103a-1 | | | | | |
| CASP5 | Caspase 5 | FADD | Fas Associated Via Death Domain | | | | | |
| AIM2 | Absent In Melanoma 2 | MEFV | MEFV Innate Immuity Regulator, Pyrin | | | | | |
| PYCARD | PYD And CARD Domain Containing | APOL1 | Apolipoprotein L1 | | | | | |
| CASP3 | Caspase 3 | TNF | Tumor Necrosis Factor | | | | | |
| DHX9 | DExH-Box Helicase 9 | VIM | Vimentin | | | | | |
| NLRP9 | NLR Family Pyrin Domain Containing 9 | CAPN1 | Calpain 1 | | | | | |
| NAIP | NLR Family Apoptosis Inhibitory Protein | JUN | Jun Proto-Oncogene, AP-1 Transcription Factor Subunit | | | | | |
| HMGB1 | High Mobility Group Box 1 | XIST | X Inactive Specific Transcript | | | | | |
| KCNQ1OT1 | KCNQ1 Opposite Strand/Antisense Transcript 1 | MIR139 | MicroRNA 139 | | | | | |
| CASP8 | Caspase 8 | ALK | ALK Receptor Tyrosine Kinase | | | | | |
| FOXO3 | Forkhead Box O3 | SIRT1 | Sirtuin 1 | | | | | |
| MALAT1 | Metastasis Associated Lung Adenocarcinoma Transcript 1 | BIRC3 | Baculoviral IAP Repeat Containing 3 | | | | | |
| IL18 | Interleukin 18 | BIRC2 | Baculoviral IAP Repeat Containing 2 | | | | | |
| APIP | APAF1 Interacting Protein | UBE2D2 | Ubiquitin Conjugating Enzyme E2 D2 | | | | | |
| TXNIP | Thioredoxin Interacting Protein | LY96 | Lymphocyte Antigen 96 | | | | | |
| GBP1 | Guanylate Binding Protein 1 | RIPK3 | Receptor Interacting Serine/Threonine Kinase 3 | | | | | |
| MIR214 | MicroRNA 214 | GLMN | Glomulin, FKBP Associated Protein | | | | | |
| CASP6 | Caspase 6 | IRGM | Immunity Related GTPase M | | | | | |
| NEK7 | NIMA Related Kinase 7 | NLRP13 | NLR Family Pyrin Domain Containing 13 | | | | | |
| GJA1 | Gap Junction Protein Alpha 1 | TUBB6 | Tubulin Beta 6 Class V | | | | | |
| P2RX7 | Purinergic Receptor P2X 7 | NOS2 | Nitric Oxide Synthase 2 | | | | | |
| MIR30C1 | MicroRNA 30c-1 | NOS1 | Nitric Oxide Synthase 1 | | | | | |
| MIR22 | MicroRNA 22 | PYDC2 | Pyrin Domain Containing 2 | | | | | |
| TP53 | Tumor Protein P53 | IFI16 | Interferon Gamma Inducible Protein 16 | | | | | |
| MALT1 | MALT1 Paracaspase | AKT1 | AKT Serine/Threonine Kinase 1 | | | | | |
| AGER | Advanced Glycosylation End-Product Specific Receptor | EGFR | Epidermal Growth Factor Receptor | | | | | |
| TET2 | Tet Methylcytosine Dioxygenase 2 | TP63 | Tumor Protein P63 | | | | | |
| MIR125A | MicroRNA 125a | ATF6 | Activating Transcription Factor 6 | | | | | |
| MIR155 | MicroRNA 155 | IRF1 | Interferon Regulatory Factor 1 | | | | | |
| EEF2K | Eukaryotic Elongation Factor 2 Kinase | IRF2 | Interferon Regulatory Factor 2 | | | | | |
| PD-L1 | CD274 Molecule | POP1 | POP1 Homolog, Ribonuclease P/MRP Subunit | | | | | |
| FGF21 | Fibroblast Growth Factor 21 | ORMDL3 | ORMDL Sphingolipid Biosynthesis Regulator 3 | | | | | |
| KLF3-AS1 | KLF3 Antisense RNA 1 | MDM2 | MDM2 Proto-Oncogene | | | | | |
| CEBPB | CCAAT Enhancer Binding Protein Beta | BTK | Bruton Tyrosine Kinase | | | | | |
| TFAM | Transcription Factor A, Mitochondrial | NFKB1 | Nuclear Factor Kappa B Subunit 1 | | | | | |
| MEG3 | Maternally Expressed 3 | STAT3 | Signal Transducer And Activator Of Transcription 3 | | | | | |
| MIR21 | MicroRNA 21 | BCL2 | BCL2 Apoptosis Regulator | | | | | |
| MIR135B | MicroRNA 135b | TLR2 | Toll Like Receptor 2 | | | | | |
| MIR485 | MicroRNA 485 | ANXA2 | Annexin A2 | | | | | |
| STK4 | Serine/Threonine Kinase 4 | IL1RN | Interleukin 1 Receptor Antagonist | | | | | |
| PRDM1 | PR/SET Domain 1 | BECN1 | Beclin 1 | | | | | |
| PRF1 | Perforin 1 | CD14 | CD14 Molecule | | | | | |
| MST1 | Macrophage Stimulating 1 | GSTO1 | Glutathione S-Transferase Omega 1 | | | | | |
| ELAVL1 | ELAV Like RNA Binding Protein 1 | IL13 | Interleukin 13 | | | | | |
| TREM2 | Triggering Receptor Expressed On Myeloid Cells 2 | CHI3L1 | Chitinase 3 Like 1 | | | | | |
| CDKN2B-AS1 | CDKN2B Antisense RNA 1 | PANX1 | Pannexin 1 | | | | | |
| MIR9-1 | MicroRNA 9-1 | LRPPRC | Leucine Rich Pentatricopeptide Repeat Containing | | | | | |
| MIR9-2 | MicroRNA 9-2 | CXCL8 | C-X-C Motif Chemokine Ligand 8 | | | | | |
| MIR9-3 | MicroRNA 9-3 | IL13RA2 | Interleukin 13 Receptor Subunit Alpha 2 | | | | | |
| MIR497 | MicroRNA 497 | IL32 | Interleukin 32 | | | | | |
| HDAC6 | Histone Deacetylase 6 | BST2 | Bone Marrow Stromal Cell Antigen 2 | | | | | |
| SQSTM1 | Sequestosome 1 | GPER1 | G Protein-Coupled Estrogen Receptor 1 | | | | | |
| IRF3 | Interferon Regulatory Factor 3 | LYST | Lysosomal Trafficking Regulator | | | | | |
| STING1 | Stimulator Of Interferon Response CGAMP Interactor 1 | CLEC5A | C-Type Lectin Domain Containing 5A | | | | | |
| HNP1 | Hypertensive Nephropathy | GAS5 | Growth Arrest Specific 5 | | | | | |
| ZBP1 | Z-DNA Binding Protein 1 | MIR223 | MicroRNA 223 | | | | | |
| PECAM1 | Platelet And Endothelial Cell Adhesion Molecule 1 | MIR15A | MicroRNA 15a | | | | | |
| DDX3X | DEAD-Box Helicase 3 X-Linked | MIR20B | MicroRNA 20b | | | | | |
| PRTN3 | Proteinase 3 | NR1H2 | Nuclear Receptor Subfamily 1 Group H Member 2 | | | | | |
| SERPINB1 | Serpin Family B Member 1 | CAMP | Cathelicidin Antimicrobial Peptide | | | | | |
|  |  | | |  |  |  |  |  |
