# Supplementary material for: A novel pyroptosis-associated gene signature for immune status and prognosis of cutaneous melanoma
Source: PeerJ. 2021 Oct 14;9:e12304. doi: 10.7717/peerj.12304 (PMC8520690; doi:10.7717/peerj.12304)
Supplement: Supplemental Information 6 [file peerj-09-12304-s006.docx]

**Supplementary Table 2**

The 218 chemotherapy drugs of FDA approved.

| Drug name | Drug name | Drug name |
| --- | --- | --- |
| METHOTREXATE | Arsenic trioxide | Topotecan |
| 6-THIOGUANINE | AZACITIDINE | Gemcitabine |
| 6-MERCAPTOPURINE | Cladribine | Bisacodyl, active ingredient of Viraplex |
| Nitrogen mustard | Mithramycin | Irinotecan |
| Allopurinol | Asparaginase | Docetaxel |
| Actinomycin D | Ifosfamide | Depsipeptide |
| Chlorambucil | Acetalax | Simvastatin |
| Thiotepa | Fludarabine | Raltitrexed |
| Melphalan | Cisplatin | Midostaurin |
| Triethylenemelamine | Isotretinoin | 7-Ethyl-10-hydroxycamptothecin |
| Dromostanolone Propionate | Teniposide | Bortezomib |
| Acrichine | Doxorubicin | Irofulven |
| Fluorouracil | Fludarabine | Temsirolimus |
| Nandrolone phenpropionate | Bleomycin | Denileukin Diftitox Ontak |
| TESTOLACTONE | Paclitaxel | Pemetrexed |
| Mithramycin | DECITABINE | Vorinostat |
| Pipobroman | Mitomycin | Estramustine |
| Cyclophosphamide | Bendamustine | Arsenic trioxide |
| Mitomycin | Etoposide | Eribulin mesilate |
| Floxuridine | Homoharringtonine | Gefitinib |
| Hydroxyurea | Mithramycin | Erlotinib |
| Uracil mustard | Tegafur | Fulvestrant |
| Dexamethasone Decadron | Parthenolide | Celecoxib |
| Mitotane | Dexrazoxane | Zoledronate |
| DACARBAZINE | Tamoxifen | Belinostat |
| Vinblastine | PENTOSTATIN | Lapatinib |
| Acetalax | RAPAMYCIN | Irinotecan |
| Cytarabine | Carboplatin | Dasatinib |
| Vincristine | Valrubicin | Everolimus |
| Megestrol acetate | Idarubicin | Pazopanib |
| tfdu | Epirubicin | Selumetinib |
| Procarbazine | Oxaliplatin | Imatinib |
| Lomustine | MITOXANTRONE | Lapatinib |
| Daunorubicin | Cytarabine | Nelfinavir |
| Daunorubicin | Mitoxantrone | Nilotinib |
| STREPTOZOCIN | Fludarabine | Olaparib |
| Calusterone | Imiquimod | Ixabepilone |
| Estramustine | Carmustine | Raloxifene |
| Vinblastine | Mithramycin | Abiraterone |
| Fluphenazine | Rapamycin | Abiraterone |
| Afatinib | Clofarabine | Sunitinib |
| Pazopanib | Vinorelbine | LEE-011 |
| Olaparib | Doxorubicin | Osimertinib |
| Depsipeptide | Vincristine | PF-06463922 |
| pralatrexate | Pipamperone | JNJ-42756493 |
| Pemetrexed | Epirubicin | LOXO-101 |
| Bosutinib | Idelalisib | brigatinib |
| Vismodegib | Topotecan | gilteritinib |
| Actinomycin D | ARSENIC TRIOXIDE | Acalabrutinib |
| Mitomycin | 6-Mercaptopurine | Sulfatinib |
| Lenvatinib | Docetaxel | umbralisib |
| Nelarabine | Vorinostat | Copanlisib |
| Crizotinib | Gefitinib | LDK-378 |
| DAUNORUBICIN | Clofarabine | LDK-378 |
| DIGOXIN | Dasatinib | Encorafenib |
| ETHINYL ESTRADIOL | Irinotecan | Cobimetinib (isomer 1) |
| Fluorouracil | VINORELBINE | Simvastatin |
| Nitrogen mustard | Copanlisib | Belinostat |
| Melphalan | Vandetanib | Cobimetinib (isomer 1) |
| 6-Thioguanine | Cabozantinib | Cobimetinib (isomer 1) |
| TYROTHRICIN | Panobinostat | LY-2835219 |
| Vinblastine | brigatinib | IPI-145 |
| Cabozantinib | Sonidegib | NMS-E628 |
| Neratinib | Sonidegib | Ponatinib |
| Axitinib | Vemurafenib | Bleomycin |
| Etoposide | Ibrutinib | Paclitaxel |
| Azacitidine | Alectinib | Rapamycin |
| Floxuridine | ARRY-162 | Teniposide |
| tepotinib | Dabrafenib | ABT-199 |
| Trametinib | Alectinib | BMN-673 |
| Palbociclib | BMN-673 | Ixazomib citrate |
| Carfilzomib | Bosutinib | Teniposide |
| Homoharringtonine | Dacomitinib |  |
