# Supplementary material for: A novel pyroptosis-associated gene signature for immune status and prognosis of cutaneous melanoma
Source: PeerJ. 2021 Oct 14;9:e12304. doi: 10.7717/peerj.12304 (PMC8520690; doi:10.7717/peerj.12304)
Supplement: Supplemental Information 7 [file peerj-09-12304-s007.docx]

**Supplement Table 3**

Baseline characteristics of CM patients in different subgroups.

| Characteristics | TCGA-CM cohort | | | GEO cohorts | | |
| --- | --- | --- | --- | --- | --- | --- |
|  | High risk | Low risk | *P* value | High risk | Low risk | *P* value |
| **Age** |  |  |  |  |  |  |
| ≤60 | 116 (51.1%) | 132 (58.15%) | 0.1574 | 55 (24.89%) | 26 (25.49%) | 1 |
| >60 | 111 (48.9%) | 95 (41.85%) |  | 82 (37.1%) | 39 (38.24%) |  |
| Unknow | --- | --- |  | 84 (38.01%) | 37 (36.27%) |  |
| **Gender** |  |  |  |  |  |  |
| Female | 82 (36.12%) | 90 (39.65%) | 0.4983 | 84 (38.01%) | 38 (37.25%) | 0.9948 |
| Male | 145 (63.88%) | 137 (60.35%) |  | 137 (61.99%) | 64 (62.75%) |  |
| **T stage** |  |  |  |  |  |  |
| T0 | 5 (2.2%) | 18 (7.93%) | 1.00E-04 | --- | --- | --- |
| T1 | 14 (6.17%) | 27 (11.89%) |  | --- | --- |  |
| T2 | 35 (15.42%) | 41 (18.06%) |  | --- | --- |  |
| T3 | 44 (19.38%) | 45 (19.82%) |  | --- | --- |  |
| T4 | 95 (41.85%) | 52 (22.91%) |  | --- | --- |  |
| Unknow | 34 (14.98%) | 44 (19.38%) |  | --- | --- |  |
| **N stage** |  |  |  |  |  |  |
| N0 | 115 (50.66%) | 110 (48.46%) | 0.4934 | --- | --- | --- |
| N1-3 | 83 (36.56%) | 93 (40.97%) |  | --- | --- |  |
| Unknow | 29 (12.78%) | 24 (10.57%) |  | --- | --- |  |
| **M stage** |  |  |  |  |  |  |
| M0 | 202 (88.99%) | 203 (89.43%) | 1 | --- | --- | --- |
| M1 | 11 (4.85%) | 12 (5.29%) |  | --- | --- |  |
| Unknow | 14 (6.17%) | 12 (5.29%) |  | --- | --- |  |
| **TNM stage** |  |  |  |  |  |  |
| Stage Na-II | 119 (52.42%) | 99 (43.61%) | 0.1591 | --- | --- | --- |
| Stage III-IV | 90 (39.65%) | 101 (44.49%) |  | --- | --- |  |
| Unknow | 18 (7.93%) | 27 (11.89%) |  | --- | --- |  |
| **Metastatic state** |  |  |  |  |  |  |
| Metastatic | 155 (68.28%) | 201 (88.55%) | 0 | 57 (25.79%) | 26 (25.49%) | 0.9594 |
| Primary | 72 (31.72%) | 26 (11.45%) |  | 27 (12.22%) | 11 (10.78%) |  |
| Unknow | --- | --- |  | 137 (61.99%) | 65 (63.73%) |  |
