# Supplementary material for: A novel pyroptosis-associated gene signature for immune status and prognosis of cutaneous melanoma
Source: PeerJ. 2021 Oct 14;9:e12304. doi: 10.7717/peerj.12304 (PMC8520690; doi:10.7717/peerj.12304)
Supplement: Supplemental Information 8 [file peerj-09-12304-s008.docx]

**Supplementary Table 4**

All significant KEGG pathways associated with risk subgroups were analyzed by GSEA.

| Pathway name | Size | FDR *P-*value | Pathway name | Size | FDR *P-*value |
| --- | --- | --- | --- | --- | --- |
| KEGG_CHEMOKINE_SIGNALING_PATHWAY | 188 | 0 | KEGG_TYPE_I_DIABETES_MELLITUS | 41 | 4.25E-04 |
| KEGG_JAK_STAT_SIGNALING_PATHWAY | 155 | 0 | KEGG_TRYPTOPHAN_METABOLISM | 39 | 4.49E-04 |
| KEGG_TOLL_LIKE_RECEPTOR_SIGNALING_PATHWAY | 102 | 0 | KEGG_MAPK_SIGNALING_PATHWAY | 266 | 6.34E-04 |
| KEGG_VIRAL_MYOCARDITIS | 68 | 0 | KEGG_INTESTINAL_IMMUNE_NETWORK_FOR_IGA_PRODUCTION | 45 | 6.13E-04 |
| KEGG_APOPTOSIS | 87 | 0 | KEGG_PANCREATIC_CANCER | 69 | 0.00119823 |
| KEGG_FC_GAMMA_R_MEDIATED_PHAGOCYTOSIS | 96 | 0 | KEGG_PRIMARY_IMMUNODEFICIENCY | 35 | 0.00155821 |
| KEGG_CYTOKINE_CYTOKINE_RECEPTOR_INTERACTION | 263 | 0 | KEGG_ASTHMA | 28 | 0.00237109 |
| KEGG_NATURAL_KILLER_CELL_MEDIATED_CYTOTOXICITY | 132 | 0 | KEGG_NEUROTROPHIN_SIGNALING_PATHWAY | 126 | 0.00386452 |
| KEGG_ANTIGEN_PROCESSING_AND_PRESENTATION | 81 | 0 | KEGG_COMPLEMENT_AND_COAGULATION_CASCADES | 69 | 0.00483011 |
| KEGG_T_CELL_RECEPTOR_SIGNALING_PATHWAY | 108 | 0 | KEGG_NON_SMALL_CELL_LUNG_CANCER | 54 | 0.00506794 |
| KEGG_CYTOSOLIC_DNA_SENSING_PATHWAY | 54 | 0 | KEGG_ALLOGRAFT_REJECTION | 35 | 0.00509909 |
| KEGG_B_CELL_RECEPTOR_SIGNALING_PATHWAY | 75 | 0 | KEGG_GRAFT_VERSUS_HOST_DISEASE | 37 | 0.00532936 |
| KEGG_LEUKOCYTE_TRANSENDOTHELIAL_MIGRATION | 116 | 0 | KEGG_EPITHELIAL_CELL_SIGNALING_IN_HELICOBACTER_PYLORI_INFECTION | 68 | 0.00533951 |
| KEGG_CELL_ADHESION_MOLECULES_CAMS | 131 | 0 | KEGG_PANTOTHENATE_AND_COA_BIOSYNTHESIS | 16 | 0.00538484 |
| KEGG_NOD_LIKE_RECEPTOR_SIGNALING_PATHWAY | 62 | 0 | KEGG_SNARE_INTERACTIONS_IN_VESICULAR_TRANSPORT | 38 | 0.0095789 |
| KEGG_PROTEASOME | 46 | 0 | KEGG_AMYOTROPHIC_LATERAL_SCLEROSIS_ALS | 52 | 0.01093585 |
| KEGG_LEISHMANIA_INFECTION | 69 | 0 | KEGG_ADIPOCYTOKINE_SIGNALING_PATHWAY | 67 | 0.01342995 |
| KEGG_AUTOIMMUNE_THYROID_DISEASE | 50 | 0 | KEGG_RENAL_CELL_CARCINOMA | 69 | 0.01661448 |
| KEGG_HEMATOPOIETIC_CELL_LINEAGE | 85 | 0 | KEGG_VEGF_SIGNALING_PATHWAY | 76 | 0.01627602 |
| KEGG_SYSTEMIC_LUPUS_ERYTHEMATOSUS | 56 | 0 | KEGG_TYPE_II_DIABETES_MELLITUS | 47 | 0.01747127 |
| KEGG_FC_EPSILON_RI_SIGNALING_PATHWAY | 79 | 0 | KEGG_PATHWAYS_IN_CANCER | 324 | 0.0207128 |
| KEGG_PATHOGENIC_ESCHERICHIA_COLI_INFECTION | 56 | 3.87E-05 | KEGG_NICOTINATE_AND_NICOTINAMIDE_METABOLISM | 24 | 0.02230028 |
| KEGG_RIG_I_LIKE_RECEPTOR_SIGNALING_PATHWAY | 70 | 3.70E-05 | KEGG_NEUROACTIVE_LIGAND_RECEPTOR_INTERACTION | 271 | 0.03788542 |
| KEGG_PRION_DISEASES | 35 | 1.35E-04 | KEGG_LYSOSOME | 121 | 0.03873639 |
| KEGG_REGULATION_OF_ACTIN_CYTOSKELETON | 213 | 3.31E-04 | KEGG_UBIQUITIN_MEDIATED_PROTEOLYSIS | 134 | 0.04134918 |
| KEGG_ACUTE_MYELOID_LEUKEMIA | 57 | 3.58E-04 | KEGG_GLIOMA | 65 | 0.04098754 |
| KEGG_ENDOCYTOSIS | 181 | 3.44E-04 |  |  |  |
