# Supplementary material for: A novel pyroptosis-associated gene signature for immune status and prognosis of cutaneous melanoma
Source: PeerJ. 2021 Oct 14;9:e12304. doi: 10.7717/peerj.12304 (PMC8520690; doi:10.7717/peerj.12304)
Supplement: Supplemental Information 9 [file peerj-09-12304-s009.docx]

**Supplementary Table 5**

Association between the identified pyroptosis genes expression and chemotherapy drug sensitivity.

| Gene | Drug | Correlation | *P* value |
| --- | --- | --- | --- |
| GZMA | Nelarabine | 0.91586741 | 1.17E-24 |
| GZMA | Dexamethasone Decadron | 0.63908152 | 3.90E-08 |
| GZMA | Fluphenazine | 0.61762005 | 1.47E-07 |
| GSDMC | Ixazomib citrate | -0.5737304 | 1.65E-06 |
| GSDMC | Midostaurin | -0.4918605 | 6.57E-05 |
| GSDMC | Bortezomib | -0.4635575 | 0.0001914 |
| GZMA | Arsenic trioxide | 0.43731083 | 0.00047612 |
| GSDMC | pralatrexate | -0.4360583 | 0.00049639 |
| AIM2 | Procarbazine | 0.430893 | 0.0005885 |
| AIM2 | Olaparib | 0.42353689 | 0.00074649 |
| GZMA | Fludarabine | 0.41664706 | 0.00092826 |
| PD-L1 | Tamoxifen | -0.4089361 | 0.00117829 |
| AIM2 | Simvastatin | 0.40223047 | 0.00144331 |
| PD-L1 | Nilotinib | -0.3981326 | 0.00163049 |
| GZMA | Cyclophosphamide | 0.39306791 | 0.0018917 |
| GZMA | Asparaginase | 0.3871069 | 0.00224663 |
| PD-L1 | Lenvatinib | 0.38348531 | 0.00249022 |
| GSDMC | Vismodegib | -0.3791829 | 0.00280997 |
| GSDMC | Gefitinib | 0.37896895 | 0.00282678 |
| PD-L1 | Dasatinib | 0.37355023 | 0.00328353 |
| GZMA | Hydroxyurea | 0.36924711 | 0.00369168 |
| GSDMC | Vincristine | -0.3689816 | 0.00371827 |
| AIM2 | tepotinib | 0.35578484 | 0.00527337 |
| PD-L1 | Zoledronate | 0.35037087 | 0.00606117 |
| AIM2 | Imiquimod | 0.34209009 | 0.00746589 |
| GZMA | Ifosfamide | 0.32760584 | 0.01061325 |
| PD-L1 | Bleomycin | 0.32551054 | 0.0111525 |
| PD-L1 | Ixabepilone | -0.3186346 | 0.01309192 |
| AIM2 | 6-Thioguanine | 0.31826764 | 0.01320313 |
| PD-L1 | Simvastatin | 0.30896529 | 0.01630656 |
| AIM2 | LEE-011 | 0.29794543 | 0.02077163 |
| GZMA | Chlorambucil | 0.29775846 | 0.02085555 |
| GSDMC | Dacomitinib | 0.29773869 | 0.02086444 |
| PD-L1 | Lapatinib | -0.2973006 | 0.02106229 |
| GZMA | DACARBAZINE | 0.2937862 | 0.02270764 |
| AIM2 | Abiraterone | 0.29346057 | 0.02286546 |
| AIM2 | Vismodegib | 0.291809 | 0.02368023 |
| GZMA | Pipobroman | 0.29016618 | 0.02451489 |
| GZMA | Melphalan | 0.28789164 | 0.02571137 |
| GZMA | Nelfinavir | -0.2858976 | 0.02680039 |
| PD-L1 | Procarbazine | 0.2856468 | 0.02694009 |
| GZMA | Sonidegib | -0.281535 | 0.0293188 |
| AIM2 | Bleomycin | 0.27962276 | 0.03048357 |
| AIM2 | Dromostanolone Propionate | 0.27798497 | 0.03151172 |
| GZMA | Carmustine | 0.27468456 | 0.03367173 |
| GZMA | Cladribine | 0.27372755 | 0.03432065 |
| AIM2 | 6-Mercaptopurine | 0.27165548 | 0.03576134 |
| GSDMC | Idarubicin | -0.2692567 | 0.03749157 |
| GSDMC | Carmustine | -0.2666478 | 0.03945154 |
| GSDMC | DAUNORUBICIN | -0.2654265 | 0.04039773 |
| GSDMC | Erlotinib | 0.26359682 | 0.04185029 |
| GZMA | Cytarabine | 0.26355929 | 0.04188053 |
| PD-L1 | Fluorouracil | -0.2616302 | 0.04345926 |
| GZMA | DECITABINE | 0.25894756 | 0.04573579 |
| GSDMC | Bisacodyl, active ingredient of Viraplex | 0.2582365 | 0.04635533 |
| PD-L1 | Vorinostat | -0.2580812 | 0.04649157 |
| GSDMC | Pazopanib | -0.2579976 | 0.04656501 |
| GZMA | Uracil mustard | 0.25775819 | 0.04677594 |
| PD-L1 | Bosutinib | -0.2564274 | 0.04796267 |
